# Oligomeric S100A4 Is Associated With Monocyte Innate Immune Memory and Bypass of Tolerance to Subsequent Stimulation With Lipopolysaccharides

**DOI:** 10.3389/fimmu.2019.00791

**Published:** 2019-04-15

**Authors:** Michel Neidhart, Agnieszka Pajak, Katerina Laskari, Niels P. Riksen, Leo A. B. Joosten, Mihai G. Netea, Esther Lutgens, Eric S. G. Stroes, Adrian Ciurea, Oliver Distler, Mariam Grigorian, Emmanuel Karouzakis

**Affiliations:** ^1^Department of Rheumatology, Center of Experimental Rheumatology, University Hospital Zurich, University of Zurich, Zurich, Switzerland; ^2^Department of Internal Medicine, Radboud University Medical Center, Nijmegen, Netherlands; ^3^Radboud Institute for Molecular Life Sciences (RIMLS), Radboud University Medical Center, Nijmegen, Netherlands; ^4^Human Genomics Laboratory, Craiova University of Medicine and Pharmacy, Craiova, Romania; ^5^Department for Immunology & Metabolism, Life and Medical Science Institute (LIMES), University of Bonn, Bonn, Germany; ^6^Institute for Cardiovascular Prevention, Ludwig-Maximilians-University, Munich, Germany; ^7^Department of Medical Biochemistry, Amsterdam University Medical Centre, Amsterdam Cardiovascular Sciences, University of Amsterdam, Amsterdam, Netherlands; ^8^Department of Vascular Medicine, Academic Medical Center, Amsterdam, Netherlands; ^9^Faculty of Health Sciences, Center of Neuroscience, University of Copenhagen, Copenhagen, Denmark

**Keywords:** trained immunity, DAMPs, S100A4 protein, epigenetic, rheumatoid arthritis

## Abstract

**Objectives:** Most DAMPs in inflammatory diseases are TLR2- and TLR4-ligands and according to the current concept, repeated stimuli would result in tolerance. Aims of the study were to verify this assumption, to investigate whether epigenetic effectors are involved and to explore the situation in rheumatoid arthritis (RA).

**Methods:** A trained immunity (TI) and tolerance protocol was established using peripheral blood monocytes from healthy donors, β-glucan and lipopolysaccharide (LPS). The training or tolerance capacities of RA-relevant DAMPs were tested.

**Results:** β-Glucan-, oS100A4-, HMBG1-, and HSP90-pretreated monocytes showed increased IL-6 responses to LPS re-stimulation. β-Glucan, oS100A and tenascin C induced training of monocytes to release more TNFα. In comparison to β-glucan, most DAMPs tested induced less TI, with exception of oS100A4. Monocytes exposed to oS100A4 showed increased IL-1β, IL-6, and TNFα in response to LPS, in spite that both stimulate TLR4. RNASEq upon β-glucan or oS100A4 revealed similar changes in chemokines/cytokines and epigenetic effectors; 17 epigenetic effectors correlated with chemokine/cytokine gene expression; PRDM8 was associated with more chemokine and cytokine transcripts. Knockdown of PRDM8 abolished TI induced by oS100A4. In RA, plasma S100A4 correlated with increased CSF2, and increased PRDM8 transcription in RA monocytes was associated with increased plasma CCL5 and IL-6, as well as therapy-resistance.

**Conclusion:** Bypass of tolerance by DAMPs might be a phenomenon as important as TI, since it could explain how chronic inflammation can be maintained in spite of an environment with multiple TLR2/TLR4-ligands. In RA monocytes, a PRDM8-dependent TI mechanism could be responsible for sustained chemokine/cytokines levels.

## Introduction

Monocytes and macrophages play a central role in the pathophysiology of inflammation. For instance, in rheumatoid arthritis (RA), activated monocytes massively infiltrate synovial tissues and produce tumor necrosis factor-α (TNFα) (1–3). Accordingly, therapies aimed at blocking this cytokine have emerged as a major tool in the treatment of RA and other inflammatory diseases (4, 5). These observations indicate the essential role of TNFα in the pathogenesis of RA. Indeed, RA synovial fibroblasts have an intrinsic ability to destroy cartilage and bone due to epigenetic changes (6), and TNFα further stimulates their aggressive behavior (7).

Abnormal activation of pattern recognition receptors (PRRs), like Toll-like receptors (TLRs) and receptors for advanced glycation end products (RAGE) by endogenous danger signals (damage-associated molecular patterns; DAMPs) as well as infectious agents (pathogen-associated molecular patterns; PAMPs) has been suggested to be crucial for perpetuating vicious inflammatory cycles (8–10). In RA, of special interest is the oligomeric form of S100A4 (oS100A4), which is a potent trigger of pro-inflammatory cytokines found in plasma of patients with RA (11–13). Various cell types, mostly activated fibroblasts, release S100A4 (11, 12, 14). The effect of S100A4 on the production of proinflammatory cytokines is mainly mediated by TLR4 (13). Similar properties in driving persistent inflammation in RA has been suggested for extracellular HSP90 (14), tenascin C (15), HMGB1) (16), biglycan (17), and citrullinated fibrinogen (18).

Recently, it was shown that innate immune cells during initial challenge with pathogens become trained and successively undergo long-term adaptive changes in their functional programs, resulting in altered gene expression and cell physiology upon re-stimulation with the pathogen (19–21). This *de facto* novel kind of immunological memory has been termed trained immunity (TI) or innate immune memory. An *in vitro* TI protocol has been developed to explore the mechanisms governing the TI phenomenon (19, 22, 23). It has been reported that CD14+ monocytes or PBMCs pre-treated with heat-inactivated *Candida albicans* or its most potent component, β-glucan, increases production of IL-6 and TNFα upon re-stimulation with different non-related microbial stimuli. Similar training properties to β-glucan has been implicated for many other PAMPs (23). Of great importance is that dependently on PRR engaged and dosage regimen applied, tolerance (i.e., decreased cytokine production) instead of training could be induced). The current concept predicts possible training upon stimulation of different receptors and pathways, while sequential stimulation of receptors triggering common pathways result in tolerance (23, 24). Training or tolerance occur depending on the stimuli, sequence of stimulation, doses and signaling pathways.

TI in relation to endogenous danger stimuli has been shown for oxidized low density lipoproteins (25) and lipoprotein A (26). Possibly, alarm signals associated with tissue damage and sterile inflammation can induce TI through epigenetic regulation of transcriptional programs. A concern regarding TI in a disease such as RA is that mostly all circulating DAMPs are recognized by TLR2 and/or TLR4. According to the concept above, it could be expected that subsequent stimulation with LPS or repeated exposure with DAMPs will result in tolerance. We report here that oS100A4 shows important training abilities and induces cellular changes allowing to bypass tolerance to subsequent TLR4 ligands, including LPS. Here, we propose that histone methylase PRDM8 plays a pivotal role in DAMPs-induced TI. Such a mechanism might be relevant in chronic inflammatory diseases. It can be hypothesized that TI and bypass of tolerance in RA might explain the association with e.g., increased risk for atherosclerosis (27). Thus, elucidation of this pathway would potentially reveal novel pharmaceutical targets.

## Materiels and Methods

### Recombinant Human Proteins and Reagents

The selection of DAMPs was done according to the literature [e.g., ref. (13–18)] and based on following criteria: (1) known receptors, (2) receptors present on monocytes/macrophages, (3) described effect on monocyte/macrophages, (4) released as alarmins from other cells/tissues, and (5) relevance to RA.

Recombinant human proteins were used: HSP90/GRP94 (Abcam), tenascin C, HMBG1, biglycan, and S100A4 (R&D Systems). In addition, purified human oS100A4 was provided by M. Grigorian (Copenhagen University, Copenhagen, DK). β-Glucan isolated from *Candida albicans* was provided by the department of internal Medicine, Radboudumc (Nijmegen, NL). Other compounds used in the current study included human fibrinogen and citrullinated fibrinogen (Cayman), muramyl dipeptide (MDP), *E. coli* lipolpolysaccharide-B5, *Salmonella typhimurium* flagellin and ATP (Sigma). All DAMPs were tested for endotoxin contamination, using the Pierce LAL Chromogenic Endotoxin Quantification Kit. SNX482 (Tocris) was diluted to 25 nM in RPMI.

### Preparation of Monocytes From Human Buffy Coats and Peripheral Blood Mononuclear Cells

**Figure 1:**
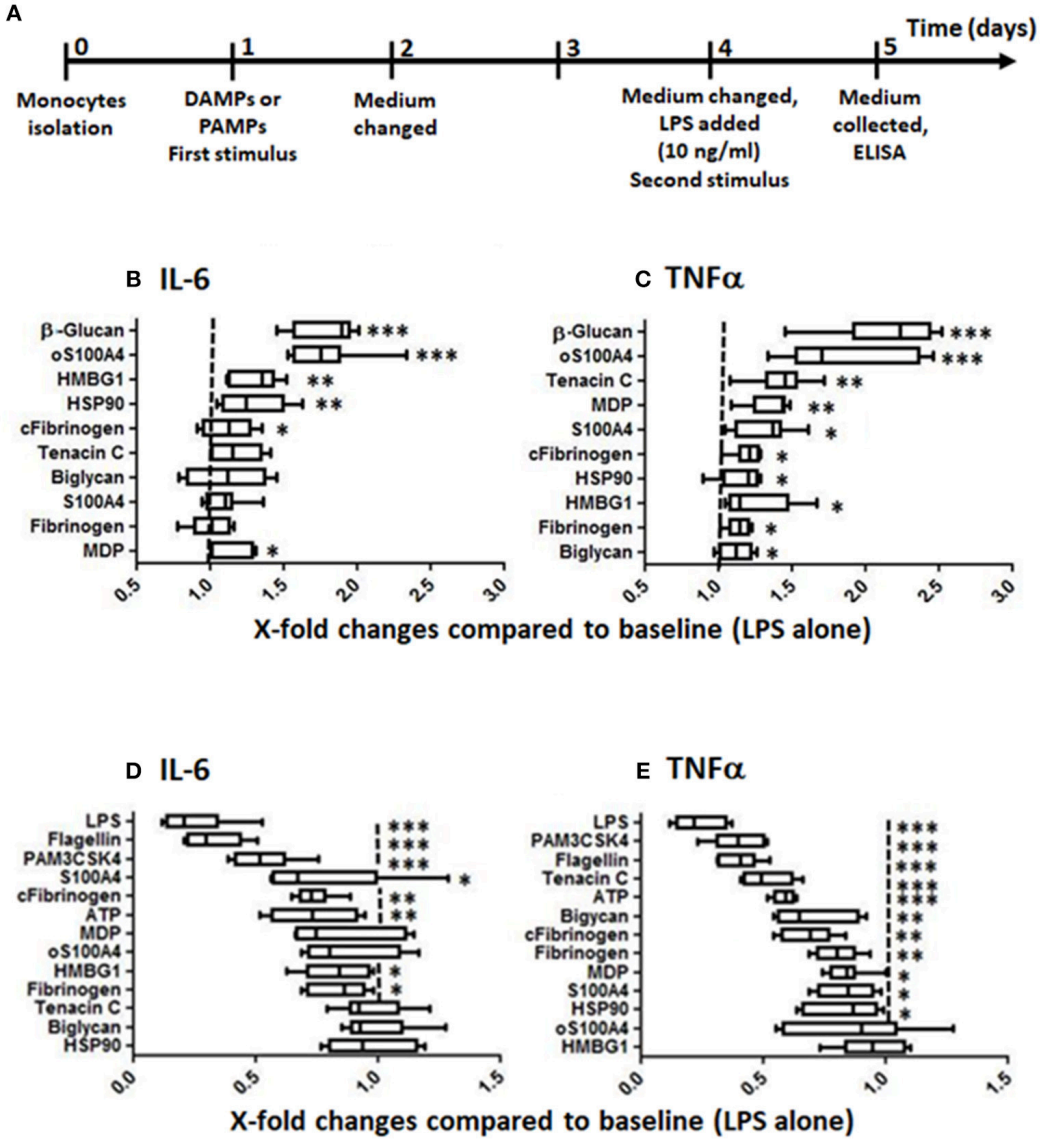
Training and tolerance induced by DAMPs. **(A)** Training/tolerance protocol. **(B,C)** Low doses of DAMPs can increase the response to LPS. **(D,E)** Higher doses of DAMPs, however, can induce tolerance to LPS. The effects were DAMPs- and read-out-dependent (box-and-whisker plots, comparison of medians using Mann-Whitney U-test, *p < 0.05, **p < 0.01, ***p < 0.001, compared to controls without first stimulus, *n* = 8 healthy donors). Optimal doses in this experiment were following: **(B,C)** training: β-glucan 1 μg/ml, biglycan 0.5 μg/ml, fibrinogen 0.15 μg/ml, citrullinated fibrinogen 0.15 μg/ml, HMGB1 2.5 μg/ml, HSP90 1.25 μg/ml, muramyl dipeptide (MDP) 1 μg/ml, oS100A 5 μg/ml, S100A4 5 μg/ml, tenascin C 1.25 μg/ml; **(D,E)** tolerance protocol: Flagellin 5 ng/ml, LPS 1 ng/ml, PAM3CSK4 0.5 μg/ml, ATP 0.31 μg/ml, biglycan 1 μg/ml, fibrinogen 1 μg/ml, citrullinated fibrinogen 1 μg/ml, HMBG1 5 μg/ml, HSP90 5 μg/ml, MDP 5 μg/ml, oS100A4 10 μg/ml, S100A4 10 μg/ml, tenascin C 5 μg/ml.

**Figure 2:**
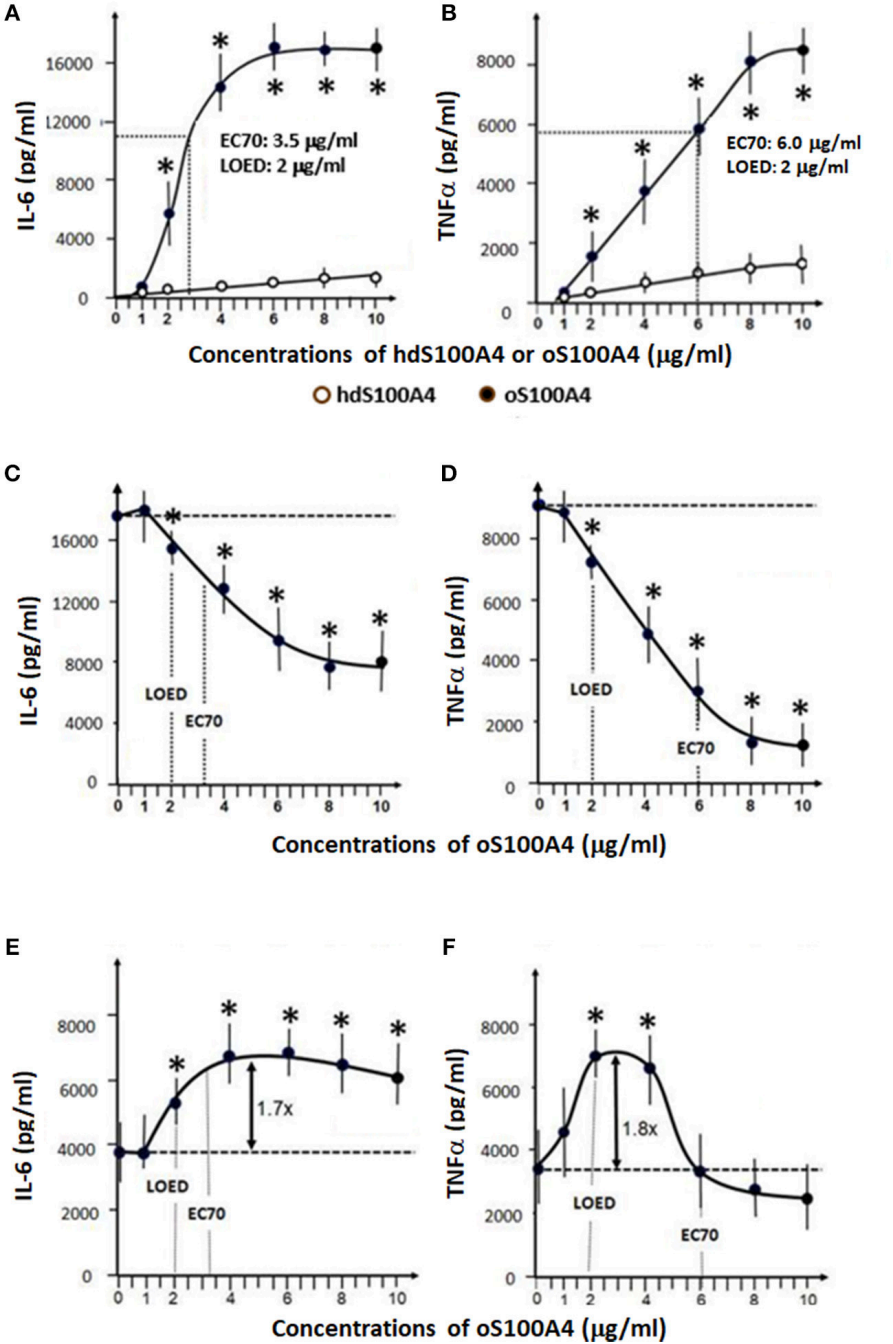
Effects of S100A4 and LPS. **(A,B)** Dose-response curves of homodimeric S100A4/oligomeric oS100A4 regarding increased releases of IL-6 and TNFα into the cell culture supernatant. **(C,D)** The effect of LPS pre-treatment (1 ng/ml) on the response to oS100A4 (1-10 μg/ml). **(E,F)** the effect of oS100A4-pre-treatement (1–10 μg/ml) on the response to LPS (10 ng/ml) (*n* = 8 healthy donors, Mann–Whitney *U*-test, *p < 0.01 vs. controls without first stimulus). LOED: lowest effective dose, EC70: refers to the concentration of a compound, which induces a response 70% between the baseline and maximum after 24 h.

In Figures 1, 2, buffy coats from healthy donors (*n* = 8, Red Cross, Schlieren, Switzerland) were diluted 5-times with PBS; 35 ml diluted blood were underlayed with 15 ml Ficoll-Paque Plus (GE Healthcare) and centrifuged at 400 g for 35 min. The cell layer (PBMCs) were washed in PBS, resuspended in RPMI+ (RPMI1640 containing GlutaMAX, HEPES, PenStrep and pyruvate) and counted. Three ml PBMCs (150 to 200 × 10^6^ cells) were layered on 10 ml 48.5% hyperosmotic Percoll solution (48.5 ml Percoll Plus [GE Healthcare] diluted in 41.5 ml sterile water and 10 ml 1.6M NaCl) and centrifuged at 580 g for 15 min. The interphase was collected, washed in PBS, and centrifuged down at 400 g for 10 min. RMPI+ were added to the cell pellet and this suspension is referred as “monocyte-enriched suspension.” The cells were counted and checked for viability.

**Figure 3:**
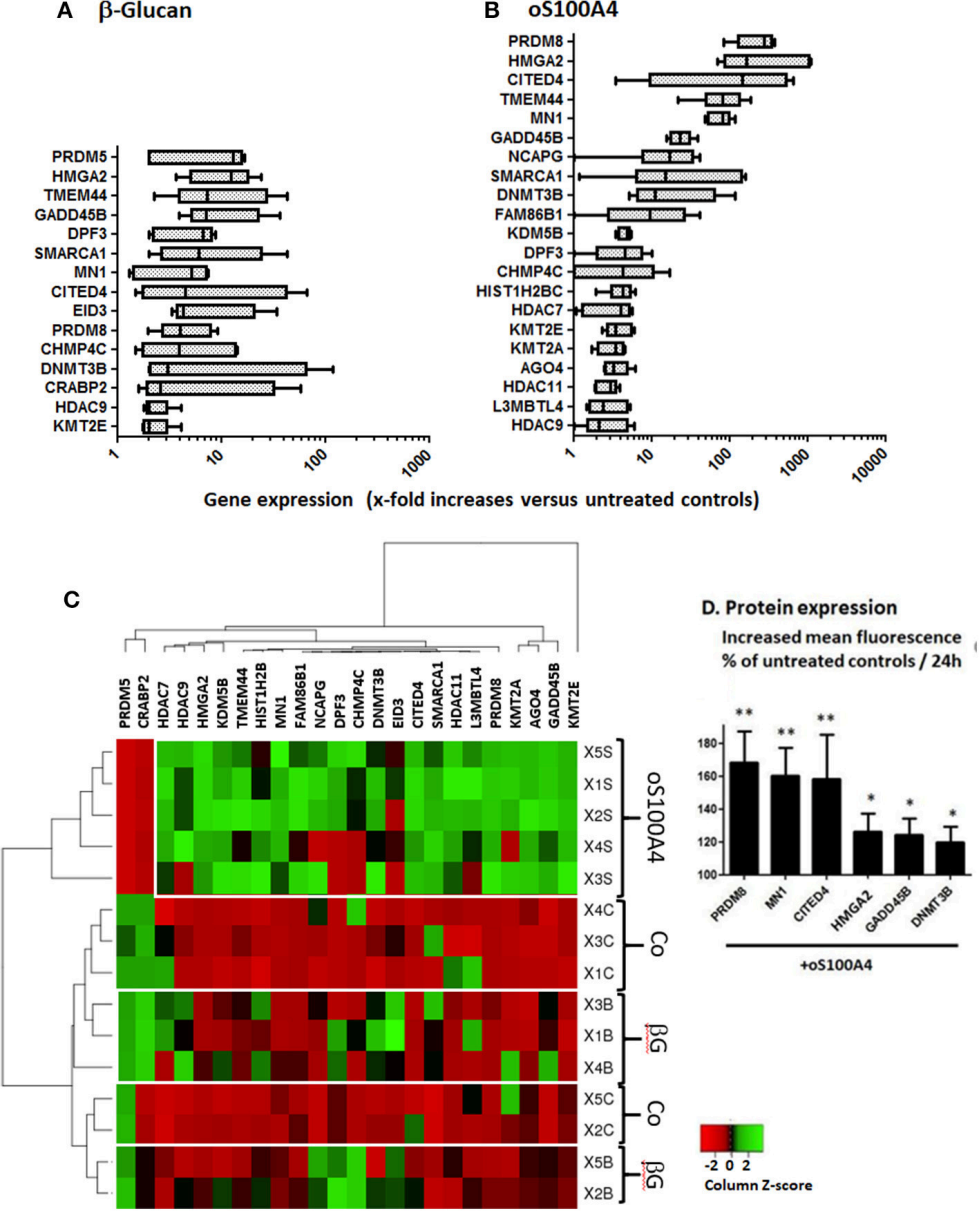
Changed gene expression of epigenetic effectors upon **(A)** β-glucan (1 μg/ml) or **(B)** oS100A4 (2 μg/ml), as revealed by RNASeq (box and whisker plots, significantly increased mean and median > 2-fold, *n* = 5 healthy donor in duplicate). **(C)** Heat map of epigenetic effectors transcripts discriminating the three conditions (controls [Co], β-glucan [βG] or oS100A4). **(D)** Increases in the cytoplasmic expression of selected epigenetic effectors in permeabilized monocytes upon oS100A4, as measured by flow cytometry (*n* = 6 healthy donors, Wilcoxon signed-rank test). The clustering was unsupervised. For each donor and transcript, a Z-score was calculated. Regarding a given transcript, the highest and lowest Z-scores are shown in green and red, respectively.

**Figure 4:**
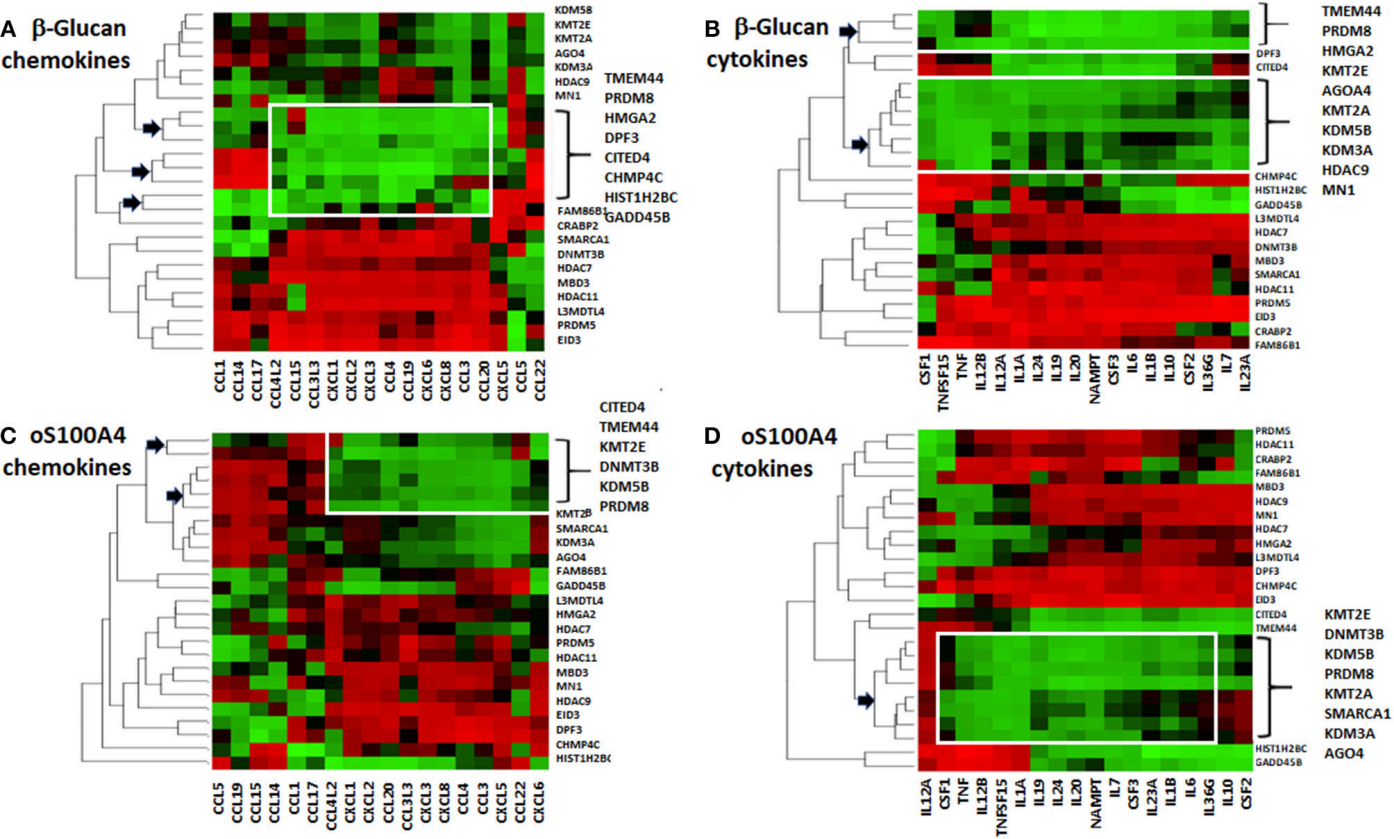
Heat maps of correlations coefficients between Z-scores of epigenetic effectors and **(A,C)** chemokines or **(B,D)** cytokines. Values for **(A,B)** β-glucan- or **(C,D)** oS100A4-stimulated cells were separately analyzed. Five healthy donors were evaluated in duplicate. The dendrogram reveals possible associations (arrows); the so-called “KMT2E/PRDM8” cluster is highlighted. Spearman's rank correlation coefficients between transcription levels of chemokines/cytokines and epigenetic effectors were obtained. For each chemokine/cytokine, Z-scores were calculated. For a given chemokine/cytokine, highest and lowest coefficients are shown in green and red, respectively. Epigenetic effectors were unsupervised and chemokine/cytokines were supervised.

**Figure 5:**
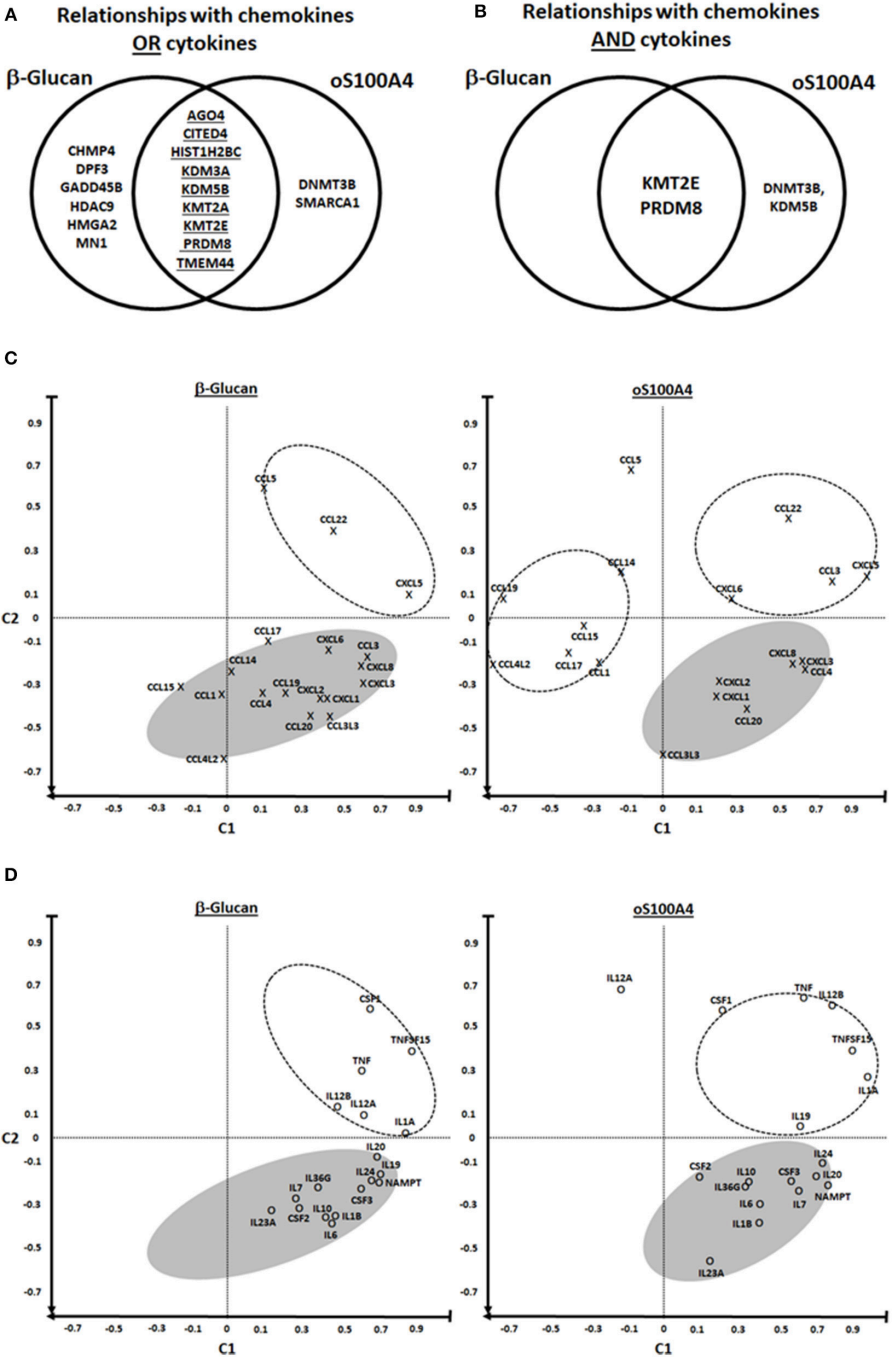
PRDM8 is a common epigenetic effector of β-glucan and oS100A4. **(A,B)** Possible relationships between chemokines/cytokines and epigenetic effectors were analyzed using Venn diagrams. The diagram left illustrates the set of 17 epigenetic effectors mRNAs which are associated with the upregulated transcription of chemokines or cytokines. The diagram right shows relationships of chemokines and cytokines, revealing KMT2E, and PRDM8 possibly as major regulators of pro-inflammatory genes in both conditions. **(C–F)** Principal component analysis revealed distinct clusters. C1 was defined as the mean correlation of a given chemokine/cytokine with KMT2E/PRDM8; C2 was defined as the mean correlation of a given chemokine/cytokine with EID3, HADC7, HDAC11, L3MTDL4, MBD3, and PRDM5 (on the heat map Figures 4A–D, they are positively associated with chemokines/cytokines outside of the so-called KMT2E/PRMD8 cluster). In the lower right quadrant are chemokines/cytokines that could be regulated by the “KMT2E/PRDM8” cluster. In the upper right quadrant are chemokines/cytokines that in addition might be modulated by other epigenetic effectors, i.e., those included in C2. In the left quadrants (particularly six chemokines upon oS100A4) are those either down-regulated and/or affected by other factors.

**Figure 6:**
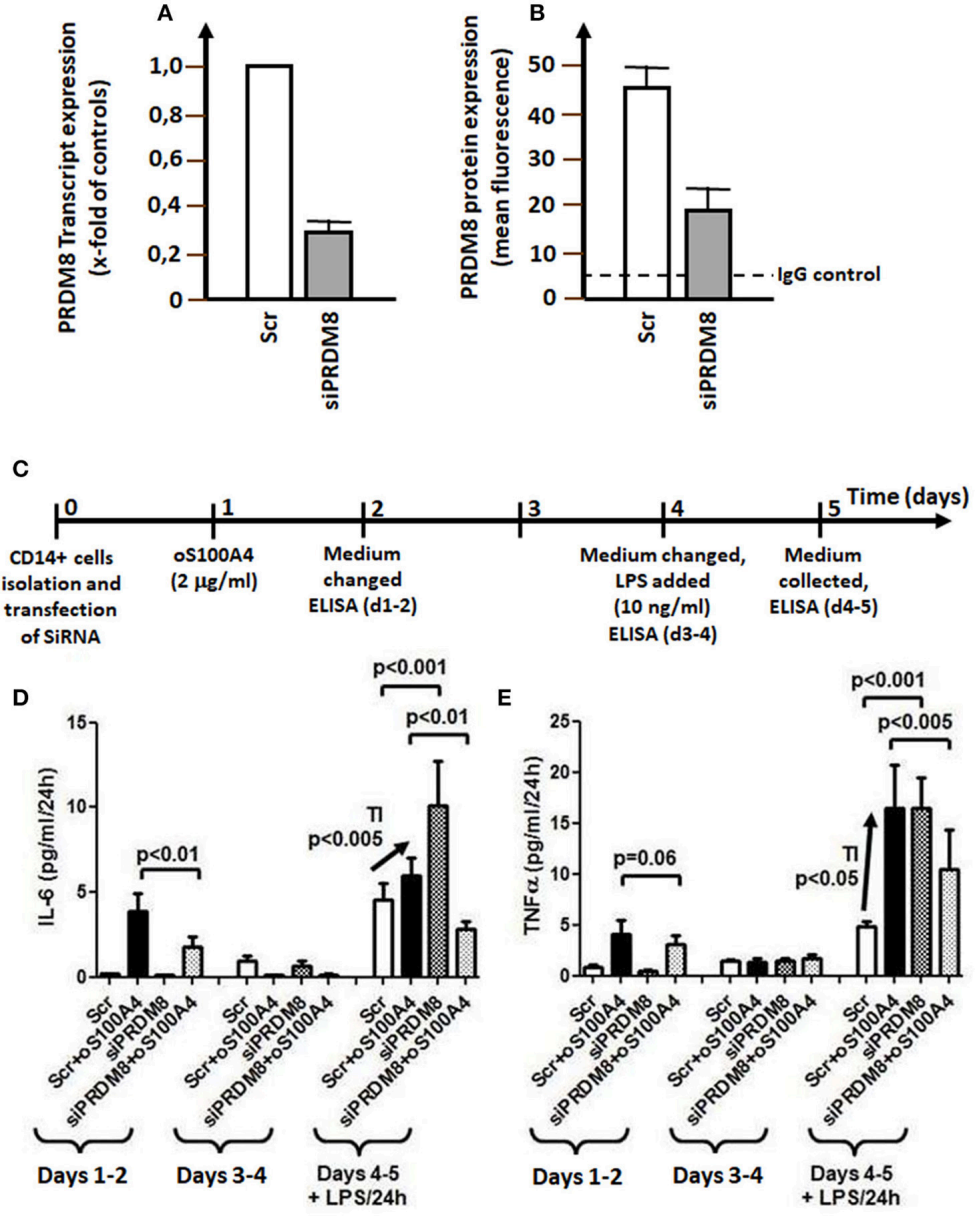
PRDM8 knockdown. **(A)** PRDM8 transcription (measured by qRT-PCR) and **(B)** PRDM8 protein expression (measured by flow cytometry in permeabilized monocytes) after 24 h. **(C)** Graphical representation of siRNA knockdown of PRDM8 in trained immunity (TI) on **(D)** IL-6 or **(E)** TNFα releases in oS100A4- (2 μg/ml) primed monocytes and subsequent stimulation with LPS (10 ng/ml) (*n* = 4 healthy donors, experiment performed in duplicate). The left panel of each figure showed the average release of cytokines during 3 days upon oS100A4, after mock transfection (scrambled, Scr) or siRNA against PRDM8. During this latency period, PRDM8 knockdown decreased oS100A4-induced release of cytokines. The middle panel shows the release of cytokines on days 4–5; at this time, the remaining effect of oS100A4 on cytokine release was negligible. The right panel illustrates the TI protocol in presence of LPS. Compared to controls (Scr), the response to LPS was significantly increased in oS100A4 (+Scr)-pretreated monocytes (arrow). sIRNA against PRDM8 increased the response to LPS in untreated cells. However, in the context of oS100A4-primed cells, the opposite occurs; TI was abolished and the response to LPS was strongly reduced.

In the other experiments (Figures 3–6, **8**), EDTA peripheral blood of healthy donors were used to isolate PBMCs by Ficoll-Paque. PBMCs were washed 2-times in cold MACS buffer, centrifuged, resuspended in RPMI+ containing 1% human serum, and counted. Monocytes were isolated by the addition of 20 μl anti-CD14 beads/10^7^ cells in 80 μl MACS buffer (Milteny Biotec). After an incubation of 15 min at 4°C, the cells were washed in cold MACS buffer, centrifuged and resuspended in 50 μl MACS buffer per 10^7^ cells, followed by magnetic separation. CD14+ cells were resuspended in RPMI+ including 1% human serum, counted and used for stimulation, as well as training and tolerance experiments.

### Monocyte Training and Tolerance Experiments

Monocyte-enriched suspensions of 2 × 10^6^ cells/ml were prepared in RPMI+ (*n* = 8). The cells were distributed in 96-wells plate (200'000 cells in 100 μl per well) and incubated at 37°C for 1 h to let them to adhere. Non-adherent cells were removed (mostly CD3+, CD14- T-cells). The cells were washed with warm PBS to remove the rest of non-adherent cells. Next, the cells were incubated in 200 ml RPMI+ including 1% human pooled serum at 37°C for 24 h. This procedure was repeated two other times and results in a population of >95% CD14+ and < 5% CD3+ cells, as checked by flow cytometry. Training was performed similarly to previous protocols (19, 22, 23). Briefly, as positive controls, monocytes were trained with 1 μg/ml β-glucan in RPMI+ containing 1% pooled human serum. RPMI+ with human serum served as a negative control. The cells were incubated for 24h at 37°C, before changing the medium. In a first set (Figure 1, Supplementary Figure 1), increasing doses of DAMPs or defined doses of PAMPs (e.g., LPS, PAM3CSK4, flagellin) allowed to differentiate between doses resulting in training or tolerance. In a second set (Figures 2A,B), monocytes were exposed for 24 h tor increasing doses of S100A4/oS100A4 (1 to 10 μg/ml). A part of the cells were pre-treated with LPS (1 ng/ml) followed by 3 days rest before re-stimulation with increasing doses of oS100A4 (Figures 2C,D). Finally, a part of the cells were pretreated with increasing doses of oS100A4 followed by 3 days rest before re-stimulation with LPS (10 ng/ml) (Figures 2E,F).

### Chemokine and Cytokine ELISA

For cell culture supernatants: IL-1β/IL-6/TNFα (BD OptEIA Human ELISA Sets, BD Bioscience), CCL5 (Quantikine R&D Systems), CXCL1 (Platinum ELISA, eBioscience) and CCL20/CSF2 (Human ELISA Kit, Thermo-Fisher), accordingly to manufacturer's protocols. For EDTA plasma: S100A4 (Aviva Systems Biology), Human IL-1β/TNFα Quantikine HS ELISA, CCL5/IL-6 Quantikine ELISA (R&D Biosystems), CSF2/CCL20/CRP ELISA (Thermo-Fisher), and CXCL1 Platinum ELISA (eBioscience).

### RNA Extraction and Quantitative Real-Time PCR (qRT-PCR)

RNA was isolated from monocytes using the RNA MicroPrep kit (Zymo Res.), according to the manufacturer's instruction; qRT-PCR was done to confirm upregulated genes and to verify the effect of siRNA. Reverse transcription was performed using 10 nM dNTP, RNase inhibitor, random hexamer, Multiscribe Reverse Transcriptase and GenAmp PCR Buffer II (all from Thermo Fisher). qRT-PCR was conducted on an Applied Biosystems 7900HT Fast Real Time PCR System, using FastStart Universal SyBr Green Master Mix (Roche) (primer pairs listed in Table 1).

**Table 1:** Primer pairs used for SYBR green quantitative RT-PCR.

CCL5 fwd 5′-CGCTGTCATCCTCATTGCTA-3′, rev 5′-GCACTTGCCACTGGTGTAGA-3′;
CCL20 fwd 5′GCGAATCAGAAGCAGCAAGC-3′, rev 5′-GATGTCACAGCCTTCATTGG-3′
CSF2 fwd 5′-AATGTTTGACCTCCAGGAGCC-3′, rev 5′-AGTGCTGCTTGTAGTGGCTG-3′;
CXCL1 fwd 5′-GAAAGCTTGCCTCAATCCTG-3′, rev 5′-CTTCCTCCTCCCTTCTGGTC-3′;
CXCL8 fwd 5′-GAAGTTTTTGAAGAGGGCTGAGA-3′, rev 5′-TCCTTGAAGTTTCACTGGCAT-3′;
IL1B fwd 5′-CTCTTCGAGGCACAAGGCAC-3′, rev 5′-TGGCTGCTTCAGACACTTGAG-3′;
IL6 fwd 5′-CCCTGAGAAAGGAGACATGTAAC-3′, rev 5′-CCTCTTTGCTGCTTTCACACATG- 3′;
TNFA fwd 5′-AGCCCATGTTGTAGCAAACCC-3′, rev 5′-TCTCTCAGCTCCACGCCATT-3′.
PRDM8 fwd 5′-CTTGCCTCCACTTCATAATGTCA-3′, rev 5′-GCTTTGACAGCCTGGTGACT-3′.
HPRT1 fwd 5′-CCTGGCGTCGTGATTAGTGA-3′, rev 5′-CGAGCAAGACGTTCAGTCCT-3′.

### Transcriptome Arrays

Five samples with 3 conditions were selected (healthy male blood donors 43 ± 11 years old). Conditions included unstimulated controls and stimulation with 1 μg/ml β-glucan or 2 μg/ml oS100A4. Adjusted volumes and cell densities for 12-well plate were 1.2 x 10^6^ cells/well and 1.2 ml medium/well. After incubation at 37°C for 24 h, cell culture supernatants were collected. Integrity of RNA was evaluated by agarose gel electrophoresis using an Agilent Bioanalyzer. RNA quality was controlled by RNA electropherograms and by using the RNA integrity number (RIN) resulting from an Agilent software algorithm. In cooperation with the Functional Genomic Center Zurich, poly A mRNA selection was performed using magnetic beads before cDNA synthesis. The cDNA libraries were sequenced using the Illumina Hiseq Sequencer. Generated were alignment QC, transcripts' expression quantification and differential expression analysis between conditions. Filtering of low read counts was applied, eliminating weakly or sporadically expressed genes. Selected were normalized genes up-regulated by >2-times (mean and median).

### Proteomic Array

The Proteome Profiler™ Human XL Cytokine Array (R&D Systems) was performed with cell culture supernatants of the transcriptome experiment, accordingly to the manufacturer's protocol. The cytokine/chemokine/growth factors profiles upon β-glucan and oS100A4 were compared.

### Flow Cytometry

CD14+ monocytes were isolated with magnetic beads as described above and incubated for 24 h with medium or 2 μg/ml oS100A4 (*n* = 6). Before staining, cells were permeabilized using Perm 2 solution (BD Bioscience). Binding of primary polyclonal rabbit anti-CITED4, HMGA2, MN1, or PRDM8 antibodies was followed by secondary staining using PE-goat anti-rabbit F(ab)_2_ (Invitrogen). Binding of murine monoclonal anti-GADD45B (Lucerna Chem.) or anti-DNMT3B (Miltenyi) was followed by secondary staining using PE-rabbit anti-mouse F(ab)_2_ (Invitrogen). Rabbit polyclonal anti-human SCIN antibodies were conjugated with FITC (LSBio). The expression was tested in THP1, PMA-differentiated THP1 cells, as well as in primary monocytes.

### Transfection

CD14+ monocytes (*n* = 4 healthy donors) were seeded into 24-wells plates. Mock or siRNA against PRDM8 transcripts (Thermo-Fisher AM16708/133027) transfections were performed using lipofectamine 2000, as described by the manufacturer (Thermo-Fisher). Two μg/ml oS100A4 were added after 24 h, corresponding to the time of maximal PRDM8 mRNA knockdown. Cell culture supernatants were collected after 3 days and the medium was replaced by RPMI+ containing 1% human serum and 10 ng/ml LPS. After 24 h of incubation, cell culture supernatant was collected and analyzed by ELISA.

### RA Plasma and Monocytes Cohort

EDTA-plasma and peripheral blood monocytes were collected from 36 RA outpatients (58 ± 10 years old) visiting the Clinic of Rheumatology at University Hospital, Zurich, as well as from 18 healthy controls (56 ± 12 years old), and RNA was prepared as previously described. The local ethical commission accepted the protocol and patients gave their informed consent (Basec 2019-0015). The samples and related clinical data were anonymized. All patients fulfilled the ACR/EULAR 2010 classification criteria for RA. In average, RA patients had an ESR of 5.0 ± 4.8 mm/h, CRP of 13.6 ± 15.1 mg/L and a DAS28 of 2.8 ± 1.2. The seropositivity for IgM-RF and anti-CCP positivity were 81 and 78% (78% were positive for both and 5% were seronegative). Treatments included Rituxumab (76% of patients), Methotrexate (41%), Tocilizumab (34%), and Leflunomid (24%). Remission was defined as a DAS28 score < 2.6, combined with BSR < 20 mm/h and CRP < 5 mg/l.

### Statistics

Mann–Whitney *U*-test was used for comparison between groups and Wilcoxon signed-rank test between control and stimulations. Spearman's rank correlation analysis allowed finding possible relationships between variables. Heat-maps using Z-scores were constructed using http://www.heatmapper.ca/expression/ after normalization to β-actin transcription. Venn diagrams were used to illustrate and select epigenetic effectors associated with chemokines/cytokines gene expressions. Two component (C1, C2) analysis was performed using means of correlation coefficients for given chemokine/cytokines and epigenetic effectors. This allowed to clearly distinguish clusters.

## Results

### DAMPs Induction of Trained Immunity

In the first set of experiments, monocytes isolated from 8 buffy coats were exposed to different doses of DAMPs and PAMPs before re-stimulation with LPS (Figure 1A). Figures 1B–E present TI or tolerance using optimal doses for IL-6 and TNFα releases. The dose ranges inducing TI were following (also shown in Supplementary Figure 1): For IL-6, oS100A4 2.5–5.0 μg/ml, HMBG1 1.25–5.0 μg/ml and HSP90 0.31–1.25 μg/ml (Figure 1B); for TNFα, oS100A4 2.5–5.0 μg/ml, tenascin C 0.31–1.25 μg/ml and MDP 0.15–1.25 μg/ml (Figure 1C). In comparison to β-glucan, most DAMPs tested produced much lower training, with exception of oS100A4. For each DAMPs and read-outs, optimal doses have to be determined.

### DAMPs and Endotoxin Tolerance

At higher doses, DAMPs can induce tolerance, i.e., reduced the response to a subsequent LPS stimulation (Figures 1D,E). Again, the effect was dose-and read-out dependent: for IL-6, citrullinated fibrinogen and ATP ≥0.31 μg/ml (Figure 1D); for TNFα, tenascin C ≥2.5 μg/ml, ATP ≥0.15 μg/ml, biglycan ≥1 μg/ml, citrullinated and native fibrinogen ≥0.61 μg/ml (Figure 1E). However, tolerance induced by DAMPs were weaker compared to repeated stimulation with PAMPs.

### Dose-Response Curves Upon S100A4 or oS100A4

In a second set of experiments using monocytes isolated from 8 additional buffy coats, we focused on the effects of oS100A4. As expected, oS100A4, compared to its homodimeric form, showed a much higher efficiency to induced pro-inflammatory cytokines, as shown by the release of IL-6 and TNFα after 24 h (Figures 2A,B).

### Sequences With LPS and oS100A4

Monocytes were pre-treated with LPS (1 ng/ml for 24 h) and 3 days later re-stimulated with increasing doses of oS100A4 (Figures 2C,D). Under such conditions, monocytes showed an oS100A4 dose-dependent induction of tolerance. Next, we investigated the dose-dependent effect of pre-treatment with oS100A4 on the subsequent response to LPS (Figures 2E,F). Monocytes were exposed to different concentrations of oS100A4, the medium was changed after 24 h, and after 3 more days, LPS was added. In contrast to the previous sequence, oS100A4-primed monocytes showed increased IL-6 and TNFα in response to LPS. Regarding IL-6, training occurred also at higher doses (4–10 μg/ml) without induction of tolerance (Figure 2E). For TNFα, a tight dose window was associated with training (2–4 μg/ml), the effect being abolished at higher doses (Figure 2F).

### Confirmation Experiments

In a third set of experiments, we investigated different combinations between β-glucan (1 μg/ml as first or 10 μg/ml as second stimulus), oS100A4 (2 μg/ml as first or 5 μg/ml as second stimulus) and LPS (1 ng/ml as first or 10 ng/ml as second stimulus). β-Glucan trained monocytes showed increased IL-6 and TNFα responses to oS100A4, similarly as they would respond to LPS. As observed in the previous sets, LPS-pre-treated monocytes induce tolerance when challenged by oS100A4. Of note, oS100A4 increased the release of IL-1β and CSF2, but these cytokines were not affected by pre-treatments with β-glucan or LPS, i.e. monocytes became not trained or tolerant for those read-outs. More important were the effects of oS100A4 as training stimulus. oS100A4-primed monocytes potentiated the effect of β-glucan, similarly to LPS-pre-treated cells. This included increases of IL-6, TNFα and IL-1β, but not CSF2 which was already maximally stimulated by oS100A4. It was confirmed that oS100A4-priming did not induce tolerance to LPS. At opposite, this sequence and combination even significantly increased the release of IL-1β and IL-6 (changes of TNFα levels in this set were not significant). oS100A4 re-challenged doesn't induce tolerance to itself (Supplementary Figure 2).

### β-Glucan-Stimulated Chemokines/Cytokines

Quantitative RT-PCR revealed upregulated transcription of CCL20, CXCL1, CXCL8, CSF2, IL1B, and IL6 by 3.5 to 12-times (Supplementary Figures 3A,B). CCL5 and TNF mRNAs were increased by 2-times only. A proteome array provided information regarding released factors after 24 h stimulation of monocytes with training doses of β-glucan (Supplementary Figure 4B). Only an increased release of CCL5 was detected. This was confirmed by ELISA.

### oS100A4-Stimulated Chemokines/Cytokines

Quantitative RT-PCR revealed upregulated transcription of numerous chemokines/cytokines, including CCL5, CCL20, CXCL1, CXCL8, CSF2, IL1B, and IL6 by 7- to 150-fold (Supplementary Figures 3A,B). TNF mRNA was unchanged. The proteome array showed that oS100A4 had a much broader effect than β-glucan, with increased releases of CCL5, CCL20, CXCL1, CXCL5, CSF2, IL-1β, and IL-6, while TNFα levels in the cell culture supernatant was only minimally changed (Supplementary Figure 4C). This was confirmed by ELISA.

### RNA Sequencing

RNASeq provided more information about global gene expression changes upon β-glucan or oS100A4. The following analysis is based on genes that were upregulated 24 h after stimulation with 1 μg/ml β-glucan or 2 μg/ml oS100A.

### oS100A4 and RNASeq

Upon stimulation of monocytes with oS100A4, a total of 1012 differentially expressed genes were divided as follow: among 902 genes with defined functions, 601 (66%) and 301 (34%) were up- or downregulate, respectively (mean and median at least > 2- or < 0.5-fold, normalized to β-actin transcription, *p* < 0.01). Functional categories with most differentially expressed genes were (in order of numbers of genes): transcription factors, growth factors and their receptors, degrading and processing enzymes, cell-cell contact and signaling, cytokines and their receptors, control of cell motility, chemokines and their receptors, extracellular matrix, G-coupled adhesion molecules and receptors, control of apoptosis and DNA repair, calcium signaling, transmembrane transporters and solute carriers, RHO-signaling, chromatin modifiers and epigenetic effectors, cell adhesion and signaling, RAS-RAF signaling, protease inhibitors, antimicrobial activities, transmembrane proteins with Ig-linked domains, ubiquitinase, and proteasome-related proteins, as well as transcription regulators and repressors. Highly upregulated in comparison to unstimulated controls were CSF2, CSF3, IL12B, PTGS2, HAS1, CYP3A5, IL24, IL20, MUCL1, TNC, IL6, IL2RA, IL19, and HHLA2. The category with most upregulated genes were transcription factors (45 genes), while the most downregulated genes were transcription regulators and repressors (18 genes). Because TI protocols used pro-inflammatory cytokines as read-outs, the analysis below will focus on chemokines/cytokines and epigenetic effectors.

### Chemokines and Cytokines

Upon β-glucan or oS100A4 stimulation (Supplementary Figures 5A,B), transcripts of 15 and 18 chemokines were significantly upregulated; 15/18 (83%) were in common between both stimuli. The difference between β-glucan and oS100A4 was more quantitative than qualitative. After both stimuli, CCL5 release was increased, as measured by ELISA (not shown). Similarly, upon β-glucan or oS100A4 (Supplementary Figures 5A–D), transcripts of 16 and 17 cytokines were upregulated; 16/19 (84%) were in common. Upon oS100A4, but not after β-glucan, CSF2, CCL20, and CXCL1 were released into the cell culture supernatant (not shown).

### Epigenetic Effectors

Upon β-glucan (Figure 3A) or oS100A4 stimulation (Figure 3B), transcripts of 13 and 22 epigenetic effectors were upregulated; 12/23 (52%) were in common. The heat map (Figure 3C) clearly showed an oS100A4-related cluster.

Epigenetics effectors with increased transcription > 7-times upon oS100A4 stimulation were confirmed at the protein level by flow cytometry. In THP-1 cells, increased CITED4 occurred upon oS100A4; other increases were obvious only in PMA-differentiated THP-1 cells, e.g., MN1, HMG2A, and PRDM8. In primary monocytes, increased expression upon oS100A4 were confirmed for CITED4, DNMT3B, GADD45B, HMGA2, MN1, and PRDM8 (Figure 3D).

Correlation heat-maps (Figures 4A–D) allowed to recognize a cluster of 17 epigenetic effectors mRNAs associated with the transcription of chemokines or cytokines (Figure 5A). The Venn diagrams allow logical operations and revealed relationships of epigenetic effectors with chemokines and cytokines (Figure 5B), suggesting that KMT2E and PRDM8 might be major regulators of pro-inflammatory mediators in both conditions. For simplification, the ranges highlighted (white) in Figure 4 are called “KMT2E/PRDM8 clusters.” Component analysis allowed using correlation coefficients to clearly distinguish clusters of chemokines/cytokines that could be associated with specific epigenetic effectors (Figures 5C–F). For instance, both IL6 and TNF belong to the KMT2E/PRDM8 cluster, but TNF might also be regulated by effectors included in C2. Upon oS100A4, some chemokines appeared in the left quadrants, suggesting that they are down-regulated by those epigenetic effectors or regulated by other factors.

Since KMT2E (MLL5) function might be more a regulator of cell cycle progression than an epigenetic effector, we focused on histone methylase PRDM8.

### PRDM8 Knockdown and Training

Twenty-four hours of after transfection, siRNA directed against PRDM8 transcripts reduced PRDM8 mRNA by 74% and intracellular protein level by 55% (Figures 6A,B). Next, we tested the effect of PRDM8 knockdown in a protocol of TI (Figures 6C,D). Transfection was performed after isolation; this was followed next day by exposure to oS100A4 for 24 h; after additional 3 days, medium was replaced including LPS. PRMD8 knockdown significantly decreased the oS100A4-induced release of cytokines over the first 3 days. Thereafter, oS100A4-primed monocytes showed significantly increased responses to LPS, reflecting a TI phenomenon. PRDM8 knockdown increased the response of control monocytes to LPS. However, in the context of oS100A4-primed monocytes, PRDM8 knockdown abolished TI in response to LPS. These data clearly associate epigenetic modulator PRDM8 with oS100A4-driven TI pathways.

### S100A4 and CSF2 in RA

**Figure 7:**
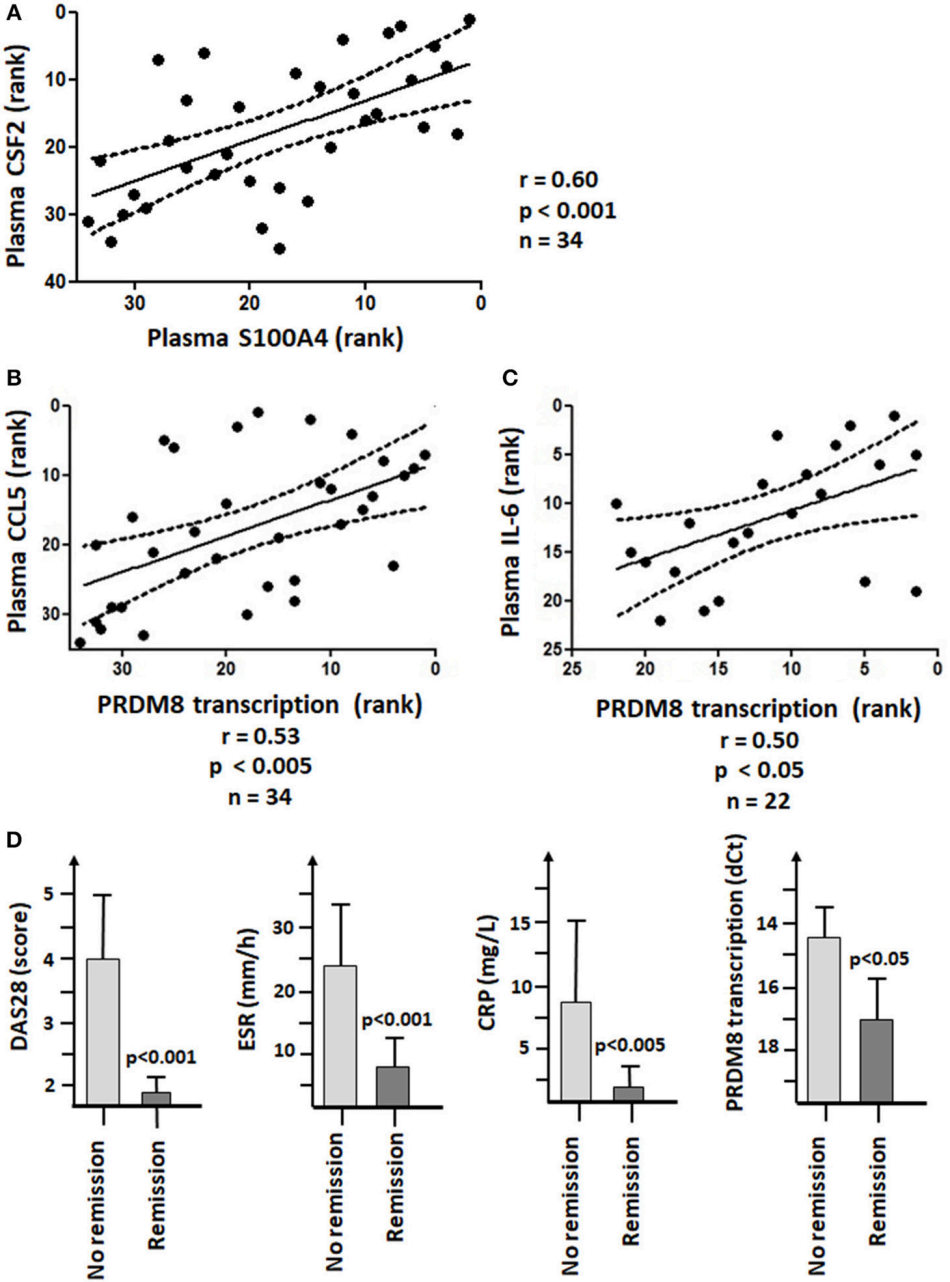
S100A4 and PRDM8 associations with the pathogenesis of RA. **(A)** Significant Spearman's rank correlation between plasma levels of S100A4 and CSF2 (GM-CSF). **(B)** Significant positive Spearman's rank correlation coefficient between PRDM8 transcription in RA monocytes and plasma levels of CCL5. **(C)** Significant positive Spearman's rank correlation coefficient between PRDM8 transcription in RA monocytes and plasma levels of IL-6 in patients not receiving Tocilizumab. The units on the X- and Y-axis corresponded to the respective ranks. **(D)** Decreased PRDM8 transcription in monocytes of patients responding to treatments or in remission (DAS28 < 2.6, BSE < 20 mm/h and CRP < 5 mg/L).

Patients with high S100A4 levels showed more CSF2 and CRP. In RA patients, a significant relationship occurred between S100A4 and CSF2 (Figure 7A).

### PRDM8, CCL5, and IL-6 in RA

qRT-PCR showed no significant difference in transcription of PRDM8 between healthy donors and RA patients. Nevertheless, in RA, PRDM8 transcription positively correlated with plasma levels of CCL5 (Figure 7B). In RA patients not receiving Tocilizumab, PRDM8 transcription positively correlated with plasma levels of IL-6 (Figure 7C). Such relationships were not observed in healthy donors. In our patient cohort, Tocilizumab was associated with higher plasma levels of IL-6 and CCL20, but lower CRP.

### Lower PRDM8 and Disease Remission

The 36 RA patients were divided into 2 groups, depending on their BSR, CRP, and DAS28 values. No remission was defined as fulfilling 2/3 following criteria: BSR > 20 mm/h, CRP > 5 mg/L and/or DAS28 score > 2.6. Taken all therapies together, poor responders showed significantly higher PRDM8 transcription (i.e., lower dCt values) than patients in remission (Figure 7D).

### Differences Between β-glucan and oS100A4

**Figure 8:**
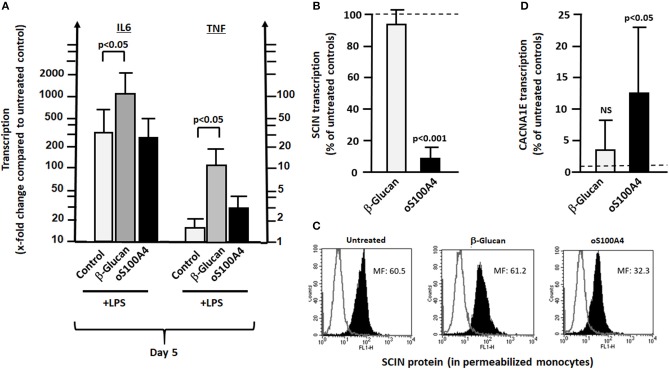
Differences between β-glucan and oS100A4. **(A)** Effect of pre-treatment with β-glucan or oS100A4 on LPS-induced transcription of IL6 and TNF (*n* = 3). β-Glucan, but not oS100A4, causes increased IL6 and TNF transcription. **(B)** RNASeq showed decreased transcription of SCIN and **C)** flow cytometry in permeabilized monocytes confirmed the reduction in scinderin (SCIN) upon oS100A4. **(D)** Increased transcription of Ca^++^ channel subunit CACNA1E upon oS100A4 (from RNASeq data).

Among differences between β-glucan and oS100A4, three important fact have to be noted (Figures 8A–D). Firstly, as expected, training with β-glucan increased LPS-induced transcription of IL6 and TNF. However, this is not the case in oS100A4-primed monocytes (Figure 8A). An alternative explanation for the increased release of pro-inflammatory cytokines in oS100A4-primed monocytes upon exposure to LPS regards modifications of the cytoskeleton, ion channels and secretory machinery. Thus, scinderin (Figures 8B,C) was downregulated, while numerous ion channels (example of a R-type Ca^++^ channel Figure 8D) were upregulated. The effect of SNX482 on THP-1 cells indeed suggested that such type of Ca^++^ channel play a role in cytokine release upon stimulation (Supplementary Figure 6).

## Discussion

### Most DAMPs Induced Weak Training

In this study, we showed for the first time that RA-related DAMPs, particularly oS100A4, can induce TI via epigenetic reprogramming involving histone methylase PRDM8. This mechanism might contribute to the well-known association between RA and e.g., cardiovascular diseases and provide a novel target for preventive pharmacotherapy.

Pre-treatment with DAMPs induced only weak training in comparison to β-glucan, with exception of oS100A4, for both IL-6 and TNFα. At higher doses, DAMPs can induce a moderate tolerance, but not comparable to endotoxin/PAMPs re-stimulations. The overall effect is dependent on the DAMP, doses and read-out.

The discrepancy between up-regulation of transcripts and missing release could be due at least in part to increased degradation in proteasomes (28, 29).

### oS100A4 Priming and Trained Immunity

As a second stimulus, oS100A4 produced similar responses as LPS. Thus, in β-glucan-trained monocytes, it increases release of IL-6 and TNFα, while in LPS-pre-treated cells releases upon oS100A4 of those cytokines were drastically inhibited. Most importantly, oS100A4-pretreated monocytes not only showed a lack of tolerance to LPS, but they even increased releases of IL-6, TNFα and IL-1β in response to LPS. A S100A4-mediated increased response to LPS can be relevant in pathogenesis (30). Of interest was the increased transcription and release of CSF2 upon oS100A4 exposure. In mice, CSF2 positively influences the TI phenomenon (31). A possibility is that CSF2 enhances TNFα production, not before but after additional LPS stimulation (32).

### Epigenetic Mechanisms and Similarities Between β-glucan and oS100A4

We performed RNASeq after exposure to β-glucan or oS100A4 and found similarities particularly for chemokines/cytokines and epigenetic effectors. Previously epigenetic reprogramming has been proposed as the molecular basis of TI (19, 33, 34). Upon β-glucan and oS100A4, our RNASeq data suggested activation of both global (HMGA2, SMARCA1) and specific epigenetic modifications. This regards upregulation of mechanisms leading to histone acetylation (CITED4, DPF3, MN1, TMEM44), deacetylation (HDAC7, 9, 11) and methylation (FAM86B1, PRDMs) as well as possibly to differential promoter methylation (DNMT3B, NCAPG) or demethylation (GADD45B).

Histone modifications are associated with transcriptional repression or activation, depending on the type of modification and locus modified. Methylation of lysine and arginine residues also can result in transcriptional activation. For instance, in rats, histone methylation is associated with persistent release of pro-inflammatory cytokines (35). Particularly interesting are the increased transcription of PR/SET domain proteins (PRDMs) encoding for histone methyltransferases. PRDMs are implicated in fundamental aspects of cellular differentiation (36, 37). Upregulated PRDM8 transcription correlated with 31 chemokines/cytokines mRNAs, including CCL5, CCL20, CSF2, IL6, and TNFA. The importance of PRDM8 is emphasized by its central position in the Venn diagram, suggesting a key role in chemokine and cytokine regulation for both stimuli. Very few biological functions are known for PRDM8, with exception that in mice fibroblasts it methylates histone H3K9 and exerts a negative regulatory role on murine steroidogenesis (38); in addition, in mice, it is required for normal development of the neocortex (39). Thus, it was important to clarify its role during the process of TI.

### Histone Demethylase PRDM8 as a Switch in Trained Immunity

PRDM8 knockdown increased the release of IL-6 and TNFα after stimulation with LPS. Thus, in the context of an endotoxin stimulation, PRDM8 may limit the pro-inflammatory response, as expected from a histone methylase. However, the situation is completely different in oS100A4-primed monocytes; siRNA against PRDM8 not only decreased the release of IL-6 and TNFα upon oS100A4, they also abolished oS100A4-induced TI. In this sequence, the effect of oS100A4 dominates over LPS, probably due to global chromatin modifications. PRDMs either act as direct histone methyltransferases or recruit a suite of histone-modifying enzymes to target promoters (37). Previously, it has been shown in mice that a histone demethylase inhibitor (but not a histone methylase inhibitor) abolished TI (19).

A limitation of the study could be the fact that monocytes from healthy donors, instead of RA treatment naïve patients were used. In patients, pre-existing chromatin changes or gene polymorphism could affect TI. Another limitation is that our analysis considers the response of monocytes to single exposure with β-glucan or oS100A4, reflecting how the cells are modified before they respond to a second stimulus. Interestingly, additional observations suggested that the mechanisms leading to an increased release of pro-inflammatory cytokines in fact are different between β-glucan- or oS100A4-pretreated monocytes. TI has been defined by increased release of pro-inflammatory cytokines into the culture supernatant and not necessarily by increased transcription. β-Glucan induces activation histone marks on the IL-6 and TNFα promoters, in particular H3K4 methylation (21). Thus, as expected, upon re-stimulation with LPS, β-glucan-trained monocytes increased the transcription of IL6 and TNF, i.e., higher than with LPS alone. This is followed by increased release. Monocytes primed with oS100A4 and re-stimulated with LPS, however, showed no more transcription of IL6 and TNF than cells exposed to LPS alone. An additional mechanism upon oS100A4 could be related to the downregulation of scinderin (SCIN), which controls the cortical membrane actin filament network. In addition, upregulation of ion channels might render monocytes more reactive to further stimuli; in particular CACNA1E, which encodes the alpha-1E subunit of the R-type Ca^++^ channels. The fact that SNX482 (a potent and selective, voltage-dependent R-type Ca^++^ channel blocker) inhibits LPS-induced release of IL-6 and TNFα (Supplementary Figure 6) suggests that upregulation of Ca^++^ channels could play a role in the increased response to a second stimulus. According to NCBI Genes, these channels mediate the entry of Ca^++^ into excitable cells and are also involved in a variety of Ca^++^-dependent processes, including muscle contraction, hormone, or neurotransmitter release, gene expression, cell motility, cell division and cell death. It is important to note that such processes also can be regulated by epigenetics (40, 41). Massive chromatin modifications occurred, as suggested by up-regulations of genes such as HMGA2, B2, or SMARCA1. Therefore, it will be interesting to investigate the effect of DAMPs on chromatin using a more global approach, such as ATAQ-Seq.

### Monocyte Training in Rheumatoid Arthritis

In RA, elevated plasma S100A4 significantly correlated with increased CSF2, in accordance with the effect of oS100A4 on CSF2 release *in vitro*. In 31% of RA patients, the plasma level of S100A4 can reach 2 μg/ml (12); in 46% of the RA synovial fluid, it ranges from 2 to 7 μg/ml. The possibility that peripheral blood macrophages in RA are trained is an important issue in the context of chronic inflammation and comorbidity with cardiovascular diseases (2, 22, 27, 42). In RA monocytes, gene expression of several proinflammatory cytokines are increased. They spontaneously showed higher releases of IL-1β and IL-6 (but not TNFα) (1). A recent study (43) reports unchanged *ex vivo* responses to LPS between healthy donors and RA patients; the authors didn't identify an increase in H3K4me3 at IL6 and TNF promoters and therefore rejected the TI hypothesis in RA. In fact, it is even surprising that RA monocytes are not tolerant to LPS, being confronted to an environment with multiple TLR2/TR4-ligands. Our data showed that DAMPs probably induced a form of TI through other mechanisms. More recently, a shift toward glycolysis has been reported in RA peripheral blood monocytes (2) that resembles trained monocytes (32, 42). In RA, the level of PRDM8 mRNAs correlated with plasma levels of CCL5; this might be a sign that DAMPs influence the activity of monocytes. Another argument in favor of the TI hypothesis in RA monocytes is the positive association between PRDM8 and plasma levels of IL-6; this is not shown by healthy donors. Both the RNASeq correlation analysis upon stimulation and the PRDM8 knockdown experiment predicted a positive association between PRDM8 and IL-6 in trained monocytes. Finally, increased PRDM8 transcription was associated with therapy-resistance.

In RA, extracellular S100A4 released by activated synovial fibroblasts is involved in the paracrine regulation of several matrix-degrading enzymes and modulation of the transcriptional activation function of the tumor suppressor protein p53 (44). In S100A4-stimulated PMBCs, enhanced production of proinflammatory cytokines is at least partly mediated by activation of the transcription factor NF-κB and the MAP kinases p38 and ERK1/2 (13). S100A4 also influences the phenotype and activity of endothelial cells (45) and vascular smooth muscle cells (46). Thus, S100A4 could be involved in the comorbidity of RA such as cardiovascular diseases.

Finally, additional studies have to investigate whether autocrine/paracrine factors, such as S100A8/S100A9 or CSF2, play a role in DAMPs-induced TI.

**Figure 9:**
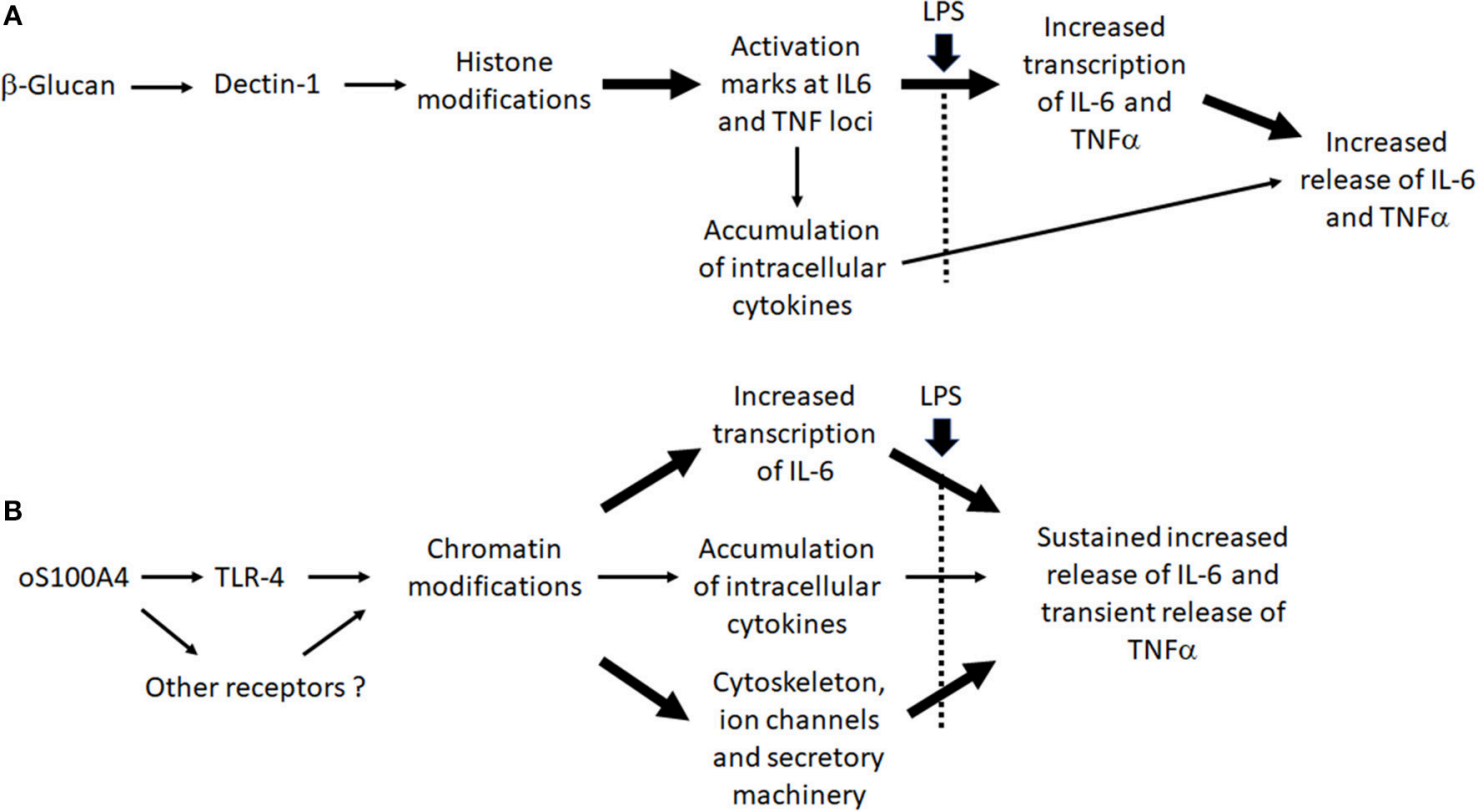
Working hypothesis: Differences between **(A)** β-glucan- and **(B)** oS100A-induced trained immunity.

In conclusion, even in the case that DAMPs act via TLR2/TLR4, this doesn't result in endotoxin tolerance. The bypass of tolerance might be a phenomenon as important as TI, since it could explain how chronic inflammation can be maintained. It also allows trained monocytes to respond adequately to infection. At concentrations measured in RA plasma, oS100A4 can induce TI. Interestingly, many similarities in chemokine/cytokine/epigenetic effectors and their relationships were found between β-glucan and oS100A4. The effect of β-glucan was explained through activating histone modifications at the IL6 and TNF loci and increased transcription upon second stimulus. This is less obvious regarding oS100A4-treated monocytes or RA monocytes. Nevertheless, the training effect of oS100A4 could be explained at different levels, including chromatin remodeling, together with changes in the cytoskeleton and secretory machinery (Figure 9). In association with epigenetic modifications, we suggested that upregulated histone methylase PRDM8 plays a key role in driving the cytokine/chemokine response. In RA monocytes, a PRDM8-dependent training mechanism could be responsible for sustained release of IL-6 and possibly other chemokines/cytokines. Through better understanding the effect of DAMPs on monocytes, novel therapeutic strategies could be developed, based on the inhibition of specific epigenetic mechanisms.

## Author Contributions

MN: conception and design of the work, analysis of data, wrote the manuscript. AP: acquisition and interpretation of data. NR, EL, and ES: design of the work, revising it critically for important intellectual content. LJ: design of the work, provided approval for publication of the content. MGN: conception and design of the work, revising it critically for important intellectual content. MG: conception and design of the work, acquisition and interpretation of data, revising it critically for important intellectual content. AC: acquisition and interpretation of data. OD: conception and design of the work, interpretation of data, revising it critically for important intellectual content. EK: conception and design of the work, acquisition and analysis of data, revising it critically for important intellectual content. KL: acquisition of data for the work, revising the new version critically, final approval. All authors provided approval for publication of the content, agree to be accountable for all aspects of the work.

### Conflict of Interest Statement

The authors declare that the research was conducted in the absence of any commercial or financial relationships that could be construed as a potential conflict of interest.

## Supplementary Material

Supplementary Figure 1 Examples of dose responses effects of DAMPs in the TI/tolerance protocol. Peripheral blood monocytes were exposed to increasing doses of DAMPs for 24 h and stimulated with 10 ng/ml LPS 3 days later (*n* = 8 healthy donors). IL-6 and TNFα were measured by ELISA in the cell culture supernatant. **(A,B)** 2.5–5.0 μg/ml oS100A4 showed TI for both IL-6 and TNFα. **(C,D)** 1.25–1.5 μg/ml HMGB1 induced TI more for IL-6 than TNFα. **(E,F)** 0.31–2.5 mg/ml tenascin C is associated with TI for TNFα only, at higher doses it produced tolerance. **(G,H)** HSP90 produced weak TI for both IL-6 and TNFα. The upper full line labeled βG represents the median TI obtained with 1 μg/ml β-glucan. The dotted line represents the baseline; the gray zone labeled LPS shows the zone of endotoxin tolerance, i.e., below the median obtained with repeated exposure to LPS (1 ng/ml followed by 10 ng/ml 3 days later).

Supplementary Figure 2 Parts of the confirmation experiment regarding repeated exposures with the same stimulus. **(A,B)** β-Glucan by itself did not result in tolerance, in contrast to the repeated exposure to LPS. At opposite, after repeated stimulation with β-glucan, a detectable increase of TNFα release can occur. **(C)** Repeated oS100A4 stimulations didn't modify the release of cytokines and most importantly didn't result in tolerance. Peripheral blood monocytes from healthy donors were exposed to 1 μg/ml β-glucan, 2 μg/ml oS100A4 or 1 ng/ml LPS for 24 h and 3 days later were re-exposed to the same stimulus. Cytokines in the cell culture supernatant were measured by ELISA.

Supplementary Figure 3 Quantitative real-time PCR. **(A)** Chemokine and **(B)** cytokine transcripts in monocytes exposed to 1 μg/ml β-glucan or 2 μg/ml oS100A4.

Supplementary Figure 4 Human XL Cytokine Array (R&D Systems)—Representative examples of cell culture supernatants: **(A)** untreated (control) CD14+ monocytes, **(B)** stimulated with 1 μg/ml β-glucan (increased CCL5, but no IL-6 or TNFα) or **(C)** 2 μg/ml oS100A4 (increased CCL5, CCL20 [MIP-3α], CSF2 [GM-CSF, CXCL1, IL-1β, IL-6, only weakly TNFα).

Supplementary Figure 5 Changed gene expression of chemokines and cytokines upon **(A,C)** β-glucan (1 μg/ml) or **(B,D)** oS100A4 (2 μg/ml), as revealed by RNASeq (mean and median > 2-fold significantly increased, box-and-whisker plots, *n* = 5 healthy donors in duplicate, Mann–Whitney U–test). **(E)** Released CCL5 and **(F)** CSF2 into the cell culture supernatant, as measured by ELISA (*n* = 10 healthy donors, Wilcoxon signed-rank test).

Supplementary Figure 6 R-type Ca^++^ channel inhibition decreased LPS-induced releases of IL-6 and TNFα from undifferentiated and PMA-differentiated THP-1 cells**. (A,B)** Within 1 h upon LPS, releases of IL-6 and TNFα from THP-1 cells into culture supernatant are inhibited by 25 nM SNX482. **(C,D)** Within 24 h upon LPS, releases of IL-6 and/or TNFα are inhibited by 25 nM SNX482, particularly by PMA-differentiated THP-1 cells (experiment in triplicate, paired *t*-test).
