# Supplementary figures and images for: Oligomeric S100A4 Is Associated With Monocyte Innate Immune Memory and Bypass of Tolerance to Subsequent Stimulation With Lipopolysaccharides
Source: Front Immunol. 2019 Apr 15;10:791. doi: 10.3389/fimmu.2019.00791 (PMC6476283; doi:10.3389/fimmu.2019.00791)

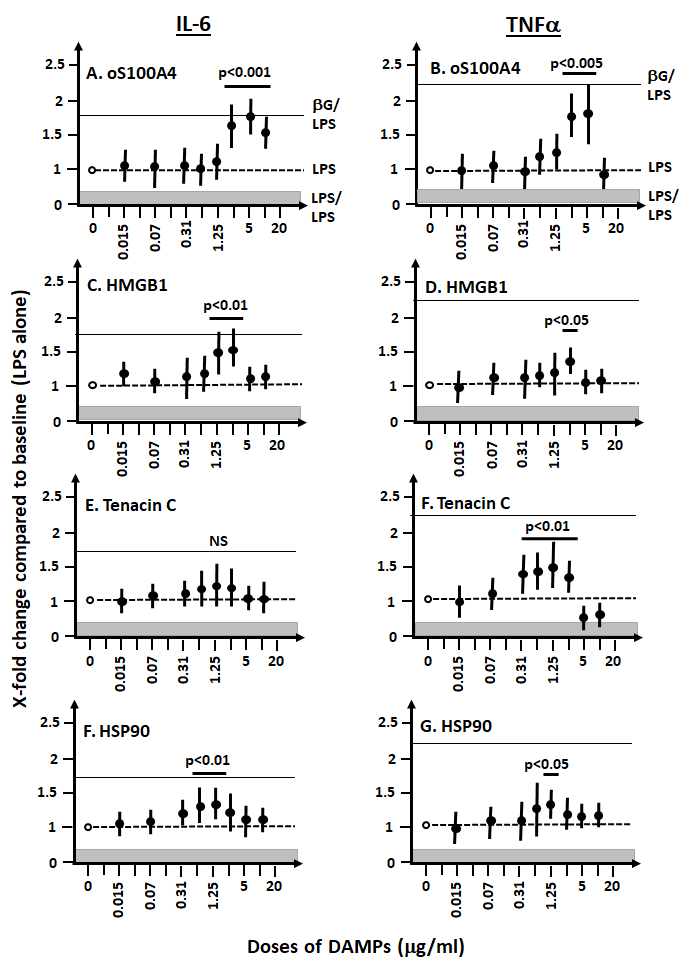

Supplement: Supplementary Figure 1 — Examples of dose responses effects of DAMPs in the TI/tolerance protocol. Peripheral blood monocytes were exposed to increasing doses of DAMPs for 24 h and stimulated with 10 ng/ml LPS 3 days later (n = 8 healthy donors). IL-6 and TNFα were measured by ELISA in the cell culture supernatant. (A,B) 2.5–5.0 μg/ml oS100A4 showed TI for both IL-6 and TNFα. (C,D) 1.25–1.5 μg/ml HMGB1 induced TI more for IL-6 than TNFα. (E,F) 0.31–2.5 mg/ml tenascin C is associated with TI for TNFα only, at higher doses it produced tolerance. (G,H) HSP90 produced weak TI for both IL-6 and TNFα. The upper full line labeled βG represents the median TI obtained with 1 μg/ml β-glucan. The dotted line represents the baseline; the gray zone labeled LPS shows the zone of endotoxin tolerance, i.e., below the median obtained with repeated exposure to LPS (1 ng/ml followed by 10 ng/ml 3 days later). [file Image_1.TIF]

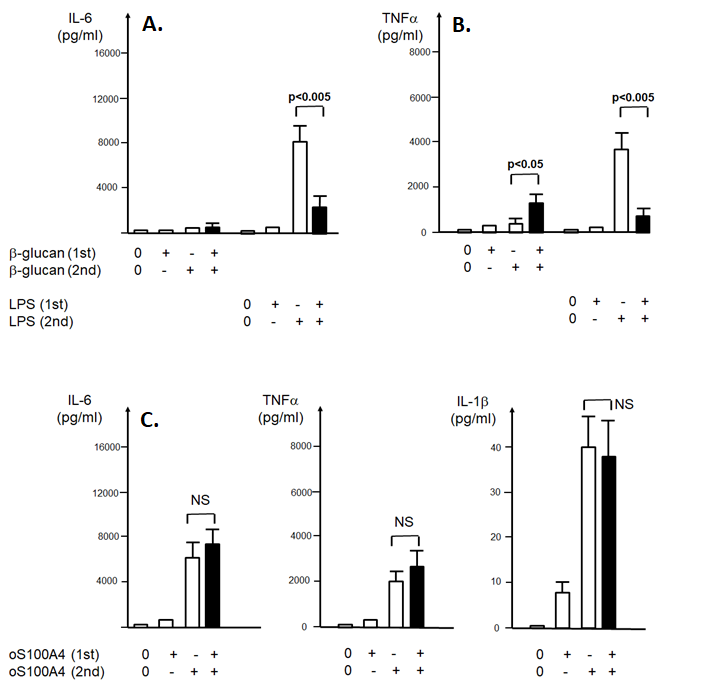

Supplement: Supplementary Figure 2 — Parts of the confirmation experiment regarding repeated exposures with the same stimulus. (A,B) β-Glucan by itself did not result in tolerance, in contrast to the repeated exposure to LPS. At opposite, after repeated stimulation with β-glucan, a detectable increase of TNFα release can occur. (C) Repeated oS100A4 stimulations didn't modify the release of cytokines and most importantly didn't result in tolerance. Peripheral blood monocytes from healthy donors were exposed to 1 μg/ml β-glucan, 2 μg/ml oS100A4 or 1 ng/ml LPS for 24 h and 3 days later were re-exposed to the same stimulus. Cytokines in the cell culture supernatant were measured by ELISA. [file Image_2.TIF]

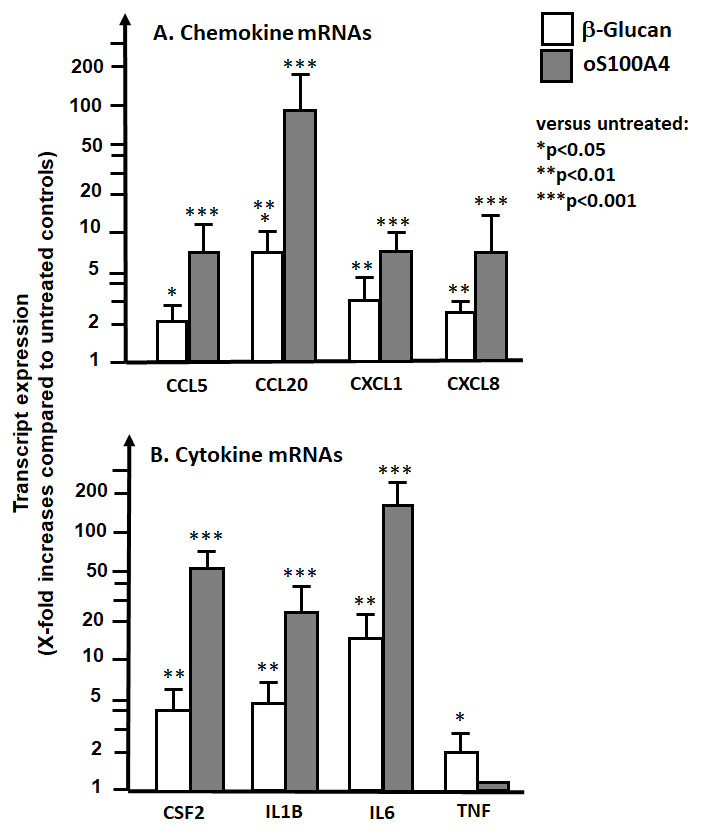

Supplement: Supplementary Figure 3 — Quantitative real-time PCR. (A) Chemokine and (B) cytokine transcripts in monocytes exposed to 1 μg/ml β-glucan or 2 μg/ml oS100A4. [file Image_3.TIF]

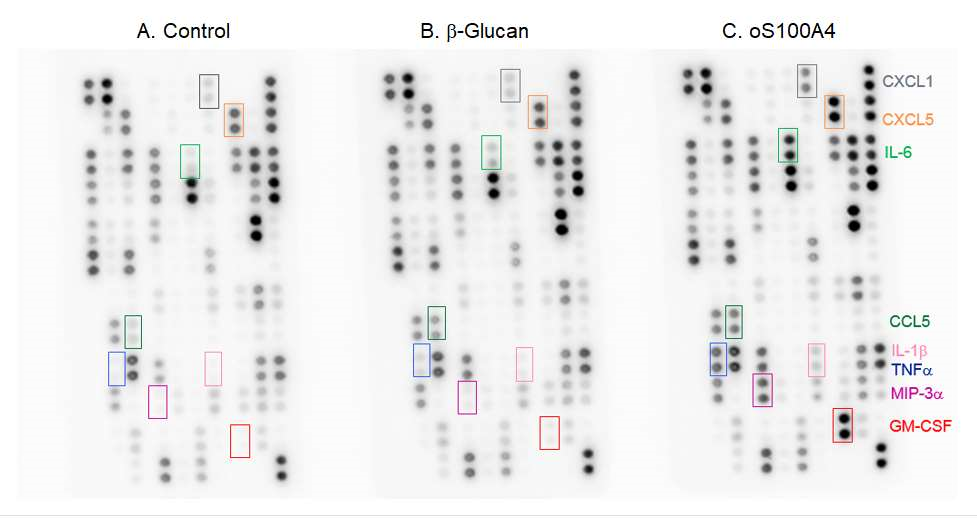

Supplement: Supplementary Figure 4 — Human XL Cytokine Array (R&D Systems)—Representative examples of cell culture supernatants: (A) untreated (control) CD14+ monocytes, (B) stimulated with 1 μg/ml β-glucan (increased CCL5, but no IL-6 or TNFα) or (C) 2 μg/ml oS100A4 (increased CCL5, CCL20 [MIP-3α], CSF2 [GM-CSF, CXCL1, IL-1β, IL-6, only weakly TNFα). [file Image_4.TIF]

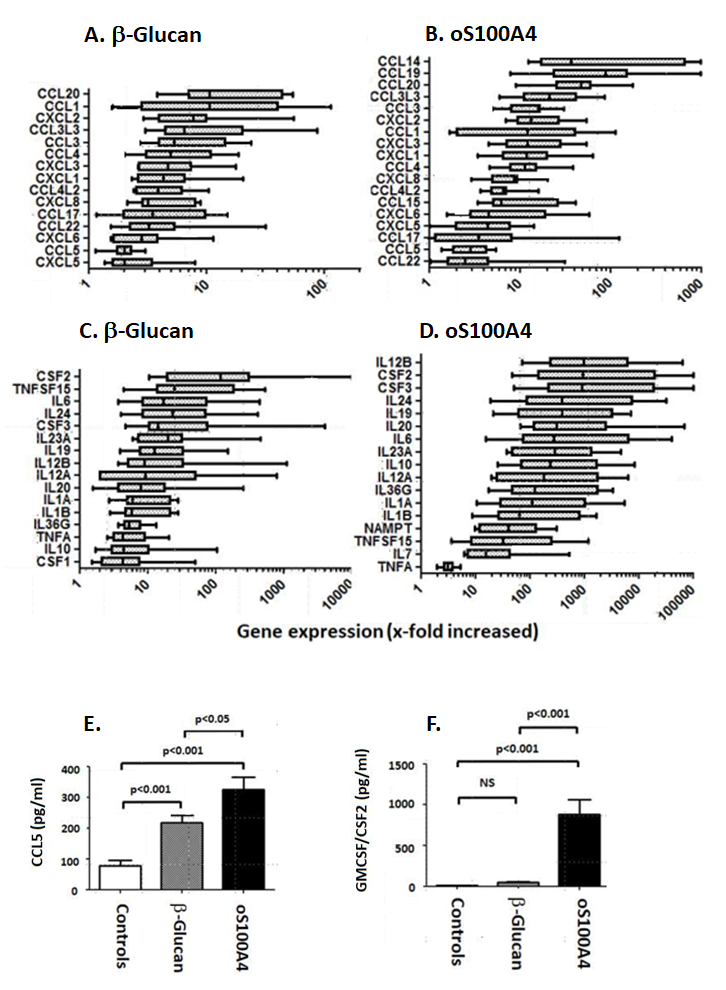

Supplement: Supplementary Figure 5 — Changed gene expression of chemokines and cytokines upon (A,C) β-glucan (1 μg/ml) or (B,D) oS100A4 (2 μg/ml), as revealed by RNASeq (mean and median > 2-fold significantly increased, box-and-whisker plots, n = 5 healthy donors in duplicate, Mann–Whitney U–test). (E) Released CCL5 and (F) CSF2 into the cell culture supernatant, as measured by ELISA (n = 10 healthy donors, Wilcoxon signed-rank test). [file Image_5.TIF]

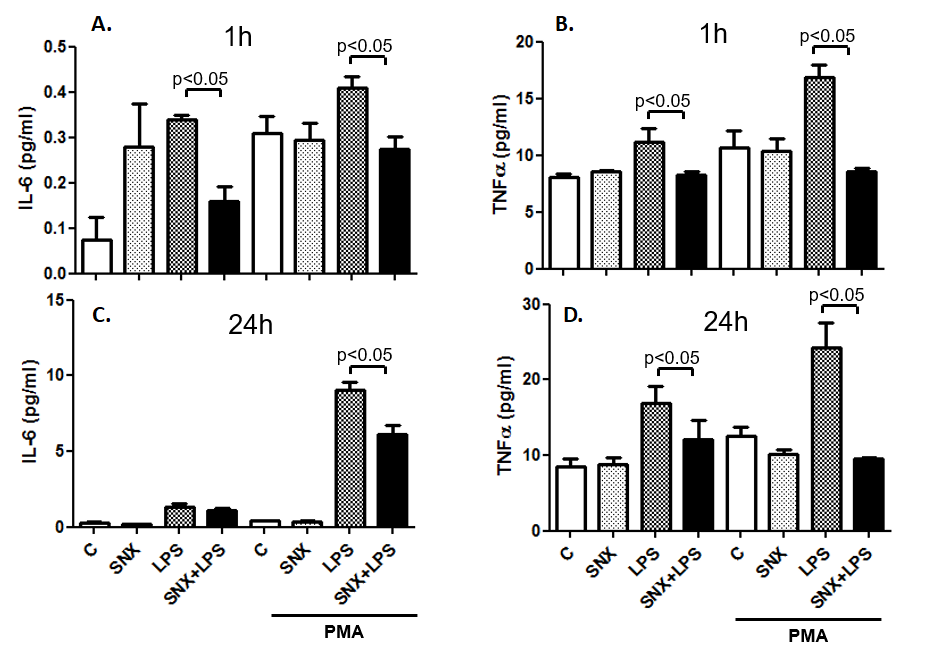

Supplement: Supplementary Figure 6 — R-type Ca++ channel inhibition decreased LPS-induced releases of IL-6 and TNFα from undifferentiated and PMA-differentiated THP-1 cells. (A,B) Within 1 h upon LPS, releases of IL-6 and TNFα from THP-1 cells into culture supernatant are inhibited by 25 nM SNX482. (C,D) Within 24 h upon LPS, releases of IL-6 and/or TNFα are inhibited by 25 nM SNX482, particularly by PMA-differentiated THP-1 cells (experiment in triplicate, paired t-test). [file Image_6.TIF]
